# Why do pregnant women present late for their first antenatal care consultation in Cameroon?

**DOI:** 10.1186/s40748-017-0067-8

**Published:** 2017-12-13

**Authors:** Paul Nkemtendong Tolefac, Gregory Edie Halle-Ekane, Valirie Ndip Agbor, Carlson Barbila Sama, Calypse Ngwasiri, Pierre Marie Tebeu

**Affiliations:** 1 Faculty of Medicine and Biomedical Sciences, University of Yaounde 1, Yaounde, Cameroon; 2 Obstetric and Gynaecology service, Douala General Hospital, Douala, Cameroon; 3 Faculty of Health Sciences, University of Buea, Buea, Cameroon; 4 Ibal Sub-Divisional Hospital, Oku, North West Region Cameroon; 5 Galactic Corps Research Group (GCRG), Buea, Cameroon; 6 Muyuka District Hospital, Muyuka, Cameroon

**Keywords:** Antenatal care, Late ANC, Booking, Determinants

## Abstract

**Background:**

Early initiation of antenatal care visits is an essential component of services to improving maternal and new born health. The Cameroonian Demographic and Health Survey conducted in 2011 indicated that only 34% of pregnant women start antenatal care in the first trimester. However, detailed study to identify factors associated with late initiation of care has not been conducted in Cameroon. The aim of this study was to assess the prevalence of late booking first ANC visit amongst attendance of first ANC and the determinants of late first ANC in Douala general hospital.

**Methods:**

It was a cross sectional analytic study over the period of 5 months in Douala general hospital. The study subjects were pregnant women visiting the facilities for the first time during the index pregnancy. Data were collected using pre-tested questionnaire. Logistic regression analysis was done to identify factors associated with late first ANC with the level of significance set at 0.05.

**Results:**

A total of 293 women participated in the study; 129 (44.0%) of them came for their first ANC visit late, after 12 weeks of gestation. Most common reasons for coming late for first ANC were financial constraints (34.5%, 45) and long distance to the hospital (34.5%, 45). Factors associated with late start of first ANC after logistic regression were: family size greater than 4 (OR = 2, 95% CI = 1.25–3.19, *p* value = 0.004), long distance to the hospital (OR = 1.84, 95% CI = 1.1–3.07, p value = 0.02) and low monthly income level less than 200US dollars (OR = 3.2, 95% CI = 1.33–3.54, p value = 0.002).

**Conclusion:**

About half of pregnant women do not start ANC early in the first trimester largely due to large family size, low monthly income and long distance to the hospital.

## Background

Globally, the maternal mortality rate (MMR) fell by nearly 44% over the past 25 years, to an estimated 216 maternal deaths per 100,000 live births in 2015. Developing regions accounted for approximately 99% (302,000) of this estimated global maternal deaths in 2015, with sub-Saharan Africa alone accounting for roughly 66% (201,000). This 2015 reports estimates MMR in Cameroon at 596 maternal deaths per 100,000 live births [1, 2]. In 2015, countries met and put forward a series of goals known as sustainable development goals (SDGs). SDG 3 calls for the acceleration of current progress in order to achieve a global MMR of 70 maternal deaths per 100,000 live births in 2030 [3]. This global reduction of MMR and a positive pregnancy outcome can only be achieved if the care offered to women during pregnancy improve and they initiates antenatal care (ANC) early enough [4, 5]. Antenatal care refers to the care that is given to an expectant mother from the time that pregnancy is confirmed until the onset of labour. This care enables the promotion of a positive pregnancy outcome. The components of ANC include: risk identification; prevention and management of pregnancy-related or concurrent diseases; and health education and health promotion [4]. Recently in 2016, WHO recommends a minimum of eight ANC contacts during pregnancy and that the first contact should be done before the 12th week of gestation [4, 6]. Planning for a safe delivery is an integral part of ANC.

According to WHO, early ANC refers to initiation of antenatal care as soon as possible after confirmation of pregnancy and or within the first 12 weeks of gestation whereas late ANC is starting ANC after 12 weeks of gestation [4]. Early initiation of antenatal care play a major role in detecting and treating some complications of pregnancy and forms a good basis for appropriate management during delivery and after childbirth. Failure to attend antenatal care early results in the potential for complications during pregnancy, delivery, and puerperium and hence increasing MMR [6, 7]. Existing evidence from most developing countries indicates that few women seek ANC services early in the course of pregnancy [8–11]. Late initiation of ANC has been associated to: maternal education, unemployment, lack of knowledge or misconceptions about the value/purpose of antenatal care, marital status, socioeconomic status, financial constraints [10]. The rate of early booking ANC visits in Cameroon is low, as is evident by the Demographic and Health Survey (DHS) report in 2011 where only 34% of pregnant women did a booking visit within the first trimester [12]. Halle et al. reported a similar prevalence of 27.2% in a health centre in Buea [13]. These findings therefore demonstrates that late initiation of ANC visit is a major public health concern in Cameroon. To the best of our knowledge, no study has been conducted to identify the determinants of late first ANC booking in Cameroon. This study seeks to determine the prevalence and determinants of late initiation of ANC among ANC attendants presenting for their first visit in Douala General Hospital (DGH).

## Methods

### Study design and setting

This was a cross sectional analytic study conducted between January and May 2017 in Douala general hospital (DGH), Cameroon. DGH is a tertiary university teaching hospital located in the economic capital of Cameroon, Douala. It is one of the fastest growing hospitals in the central African sub region. This Hospital has a service of obstetrics and gynaecology. This service runs antenatal care (ANC) outpatient clinic with a team made up of seven obstetricians and gynaecologists. The ANC clinic receives patients from Douala and its environs including referral cases from other hospitals.

### Participants

All consenting participants presenting for their first ANC visit with confirmed pregnancy were consecutively recruited, irrespective of the gestational age. Participants whose gestational age could not be determined, and those who refused to give their informed consent were excluded from the study.

### Variables

Data were collected using structured questionnaire, which was developed based on the Cameroonian Demographic and Health Survey (DHS) data collection tool and other relevant literature. The questionnaire was first developed in English and later translated into French. The information collected included socio-demographic background of mothers (e.g. age, marital status, level of education, distance from the hospital, income level, and profession obstetric history (gravid status, parity, gestational age) and reasons for presenting late. After collection, the data was entered into epi-data and then exported to SPSS version 20.0 for analysis.

### Sample size and sampling

The minimum sample size was calculated using the Lorenz formula [14] and the prevalence of women who started ANC late after 12 weeks in Cameroon at 66% according to the 2011 Cameroonian DHS [12], this minimum sample size was estimated at 345 participants. In this index study described herein, the consecutive and convenient non probability sampling technique was used where all pregnant women presenting for their first antenatal care were recruited.

### Statistical methods

This data was analysed using SPSS version 20.0. Continuous variables were presented are as means and standard deviations and categorical variables as frequencies and percentages. The proportion of late first ANC attendants was determined and the 95% confidence interval of the main outcome variable was calculated to show the extent of late initiation of ANC visit at the cut-off point of 12 weeks. The association of independent variables with late ANC initiation was examined by calculating the odds ratios. Nine variables were examined independently using a bivariate analysis.

### Ethical considerations

Ethical and administrative approval were obtained from Douala general hospital. Written informed consent was obtained from all those who participated in the study.

## Results

**Fig. 1:**
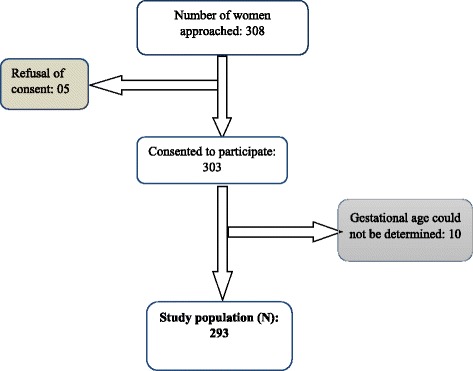
Flow chart showing recruitment of participants

We approached a total 308 women during their booking ANC. Fifteen women were excluded as shown on the recruitment flow chart on Fig. 1 below. We finally retained 293 women for the study. The mean gestational age at booking was 14 weeks ± 1.2 weeks. While Table 1 shows the baseline characters of women attending ANC in this hospital, Table 2 shows reasons these women frequently announced for late initation of ANC.

**Table 1:** Baseline characteristics of the study population

Category	Characteristics	Frequency (*N*)	Percentage (%)
Age distribution	16–25	83	28.3
	26–35	180	61.4
	36–45	30	10.3
Marital status	Married	114	38.9
	Single	179	61.1
Level of education	No formal education	6	2.0
	Primary	25	8.5
	Secondary	118	40.3
	University	144	49.1
Occupation of woman	Employed	130	44.4
	Not employed	163	55.6
Occupation of partner	Employed	271	92.5
	Not employed	22	7.5
Gravid status	Primigravida (1)	106	36.2
	Multigravida (2–4)	152	51.9
	Grand multigravida (≥ 5)	35	11.9
Parity	Nullipara (0)	126	43.0
	Paucipara (1–2)	124	124.0
	Multipara (3–4)	38	38.0
	Grand multipara (5)	5	1.7

The mean age at presentation for first ANC was 27.8 ± 5.4 years with about a 3/5 of the participants 26–35 years. Table 1 shows the distribution of baseline sociodemographic and obstetric characteristics of participants at presentation

**Table 2:** Reasons of late first ANC in the 129 participants who started booking ANC after 12 weeks

Reason	Frequency	Percentage
Financial difficulties	45	34.5
Busy schedule	25	19.4
Uncertainty of pregnancy	9	7.0
Fatigue	3	2.3
Unawareness of gestational age	2	1.6
Laziness	8	6.2
Thought this was the right time to come	12	9.3
Long distance to the hospital	45	34.5
In apparent good health	18	14.0

**Fig. 2:**
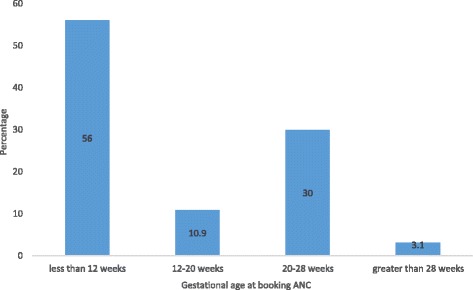
Classification according to gestational age at booking

The mean gestational age at booking was 14 weeks ±1.2 week. Amongst the 293 first ANC attendants, 129 participants (44.0%) started their booking ANC late (after 12 weeks of gestation). As shown on Fig. 2, for those who started their first ANC late, 68.2% (30.0% of total) started between 20th -28th of gestation. The commonest reasons of late first ANC advanced by these 129 participants were financial difficulties (34.5%, 45), long distances to the hospital (34.5%, 45) and busy schedule (19.4%, 25). Other reasons advanced by these women for late initiation of ANC are as shown on Table 2

### Determinants of late first ANC

As shown on Table 3, the determinants of late first ANC included: family size greater than 4 (OR = 2, 95% CI = 1.25–3.19, *p* value = 0.004), long distance to the hospital (OR = 1.84, 95% CI = 1.1–3.07, p value = 0.02) and low monthly income level less than 200US (OR = 3.2, 95% CI = 1.33–3.54, *p* value = 0.002).

**Table 3:** Determinants of first ANC using univariate logistic regression

Category	Characteristic	First ANC		OR (95% CI)	*p* value
		≤ 12 weeks (*N* = 164)	> 12 weeks (*N* = 129)		
		Number (%)	Number (%)		
Age distribution	16–25	53 (32.3)	30 (23.3)	1	
	26–35	92 (56.1)	88 (68.2)	1.21 (0.75–1.96)	0.430
	36–45	19 (11.6)	11 (8.5)	0.71 (0.45–1.11)	
Marital	Married	64 (39.0)	50 (38.8)	1	
	Single	100 (61.0)	79 (61.2)	0.99 (0.62–1.59)	0.963
Level of education	Primary	14 (8.5)	11 (8.5)	1	
	No formal education	1 (0.6)	5 (3.9)	0.80 (0.43–1.52)	0.350
	Secondary	62 (37.8)	56 (43.4)	1.26 (0.79–2.02)	0.330
	University	87 (53.1)	57 (44.2)	0.701 (0.44–1.11)	0.132
Occupation of woman	Not employed	82 (50.0)	81 (62.8)	1.27 (0.69–2.35)	0.445
	Employed	82 (50.0)	48 (37.2)	1	
Gravid status	Multigravida (2–4)	79 (48.2)	73 (56.6)	1	
	Primigravida (1)	63 (38.4)	43 (33.3)	0.80 (0.50–1.30)	0.369
	Grand multigravida (≥5)	22 (13.4)	13 (10.1)	0.32 (0.35–1.50)	0.383
Parity	Multipara (2–4)	22 (13.4)	16 (12.4)	1	
	Nullipara (0)	77 (47.0)	49 (38.0)	0.69 (0.43–1.11)	0.124
	Paucipara (1)	63 (38.4)	61 (47.3)	1.44 (0.90–2.30)	0.128
	Grand multipara (≥5)	2 (1.2)	3 (2.3)	1.93 (0.32–11.72)	0.476
Family size (F)	F < 4	98 (59.8)	55 (42.6)	1	
	F ≥ 4	66 (40.2)	74 (57.4)	2 .0 (1.25–3.19)	0.004
Average monthly income (I)	*I* ≥ 200USD	142 (86.6)	71 (55.0)	1	
	*I* < 200USD	22 (13.4)	58 (45.0)	3.2 (1.33–3.54)	0.002
Distance to the hospital (D)	D < 10 km	127 (77.4)	84 (65.1)	1	
	D ≥ 10 km	37 (22.6)	45 (34.9)	1.84 (1.1–3.07)	0.020

## Discussions

The result of the cross sectional study among ANC attendants in DGH who presented for their booking visit show that 44.0% presented late. The most common reasons advanced for late start of first ANC were financial difficulties, busy schedules and long distance to the hospitals. Three factors were associated with late first ANC: large family size, distance to the hospital. The prevalence of late first ANC amongst first ANC attendants of 44.0% means that almost half of pregnant women in this reference hospital starts their ANC late. This contradicts findings gotten earlier by Halle et al. [13] in a rural health centre in Cameroon where only 27% started their first ANC during the first trimester versus 73% that started their first ANC late [13]. The difference could be explained by the fact that Halle et al. conducted their study in a health centre located in a semi-urban area (Buea) whereas we conducted our study in a reference hospital in an urban area (Douala) as the community in Douala may have better awareness compared to the community in Buea. The very high prevalence of 73% gotten by Halle et al. [13] could also be explained by the fact that their prevalence of 73% was gotten amongst all ANC attendants whereas ours was only amongst first ANC attendants. The results are also different from the 2011 report of the Cameroonian DHS where the percentage of pregnant women who started their first ANC during the first trimester was 34% versus 66% who started their first ANC late [12]. This could be explained by the five-year time difference between the two studies and hence a possible amelioration. The results are similar to those obtained recently in 2016 and 2017 by authors elsewhere in sub-Sahara Africa where the prevalence of late first ANC ranges between 42 and 56% [6, 15, 16].

Varied reasons were advanced for late antenatal booking including financial constraints, long distances to the hospital, and busy schedule with the commonest reason being financial constraints (34.5%) and busy schedule (34.5%). These reasons are similar to those in a study conducted in 2010 and 2017 in Nigeria and Ethiopia respectively where financial constraints where amongst the commonest reasons for late first ANC [11, 16]. Other reasons noted in other series but not found in our study included reasons such as she started ANC elsewhere [9]. The mean gestational age at first ANC attendance of 14 ± 1.2 weeks means that most of the women present late for first ANC. Considering, that the first booking ANC is very important f or localisation of pregnancy, screening of common anomalies and prevention of neural tube defects with folate, these minor anomalies may be missed as the mean gestational age of presentation is after the window period of 8–12 weeks. This mean gestational age is similar to 15.9 ± 3.7 weeks and 17 ± 5.3 weeks obtained by Girum et al. and Sh et al. respectively [6, 17].

The odds of starting ANC late was about 2 times higher if the participant live greater than 10 km from the hospital. This signifies that the further the patient lives away from the hospital, the more likely is the patient to start the first ANC late. This means patients in rural areas that are more likely to walk for long distances before getting to the hospital will most of the time present late for the first ANC. Recent studies in sub-Sahara Africa earlier demonstrated that the odds of coming late for the first ANC were 2–5 times higher if the patient came from a rural area [6, 8, 18]. Also, the odds of coming late for first ANC was two times if the family size was greater than or equal to 4 persons. This means that as the size of the house gets larger the likelihood of attending ANC early drops. This can be explained by financial constraints which increases as the family size increases. This was earlier demonstrated in this study as about a third of those who came late their reasons were due to financial constraints. Recent series in sub-Sahara Africa have demonstrated similar results where the odds was 2–4 times if the family size was 3–5 [6, 8, 18]. The odds of starting first ANC late were 3 times higher if the woman had a monthly income less than 200 US dollars. 200 US dollars equals about 100,000 FRS which is the minimum amount of money needed for ANC registration and the initial prenatal investigations. This value is far above the minimum wage in Cameroon of 28.000 FRS per month. This signifies that most people whose wages in Cameroon are far above the minimum wage may still start ANC late. This is further confirmed by the fact that in this present study about a third of those who presented late had financial constraints. This is similar to studies obtained earlier in Ethiopia by Girum et al. [6] and Gebremeskel et al. [8] where monthly low income level was significantly associated with starting late starting of first ANC [6, 8].

Even though this study has come up with important finding with respect to late registration for first ANC visit, there are certain limitations worth mentioning here. Due to cross- sectional nature of the study temporal relationship could not be ascertained. The other concern was pregnant women who attended ANC at private health facilities and in rural areas are not included in the study. Moreover, gestational age was determined based on women’s reports of their last menstrual period. In addition, our small sample size and single centre study limits it application. We therefore recommend that a larger multicentre cohort study be conducted to better describe the determinants of late first ANC attendance.

## Conclusion

The study showed that nearly half of women starts their first ANC booking later than the WHO recommended time. Financial constraints and long distances to the hospital were major reasons advanced by about a third of those who presented late during the first ANC. Leaving more than 10 km away from the hospital, having a family size greater than or equal to 4 persons and having a lower monthly income less than 200 US dollars were factors significantly associated with late first antenatal care booking. Therefore addressing and empowering women through improving economic capacity and effective family planning are important measures to curb the problem.

## Abbreviations

ANC: Antenatal care
DGH: Douala General Hospital
MMR: Maternal mortality rate
